# Linear Drive of SPIONs Using Moving Magnetic Fields

**DOI:** 10.3390/mi17101125

**Published:** 2026-09-27

**Authors:** Malarvizhi Ganesan, Georg Fischer, Angelika S. Thalmayer

**Affiliations:** Institute for Smart Electronics and Systems, Friedrich-Alexander-Universität Erlangen-Nürnberg, 91054 Erlangen, Germany; georg.fischer@fau.de (G.F.); angelika.thalmayer@fau.de (A.S.T.)

**Keywords:** SPIONs, drug delivery, linear motor, MAGLEV, MAGLEV trains, MNP, speed synchronization, particle steering, magnetic nanoparticles

## Abstract

Superparamagnetic iron oxide nanoparticles (SPIONs) are widely used in many applications, from lab on chips to magnetic drug targeting to recycling batteries. SPIONs are coated with drugs and steered to reach tumor cells or are used in lab-on-chips for magnetic separation. In this paper, the SPIONs’ movement is controlled in such a way that, when the flow speed of the background fluid and the speed of moving magnetic field are synchronized, the SPIONs can be navigated to the target region more efficiently. This principle is inspired by linear motors and the propulsion principle of magnetic levitation trains, where the speed of the train is synchronized with the moving magnetic field by the static coils.

## 1. Introduction

SPIONs —Super paramagnetic iron oxide nanoparticles are the basis of an evolving technology for magnetic drug targeting in the medical domain. They have been studied for their nanoscale size and have been designed for various applications [1,2] especially in biomedical applications. Various research studies have been performed on magnetic drug targeting, hyperthermia, and potential cancer treatments. This paper describes SPIONs and how they can be steered to their target region to accomplish a desired task.

When it comes to using drug carriers, extensive research has been carried out in this area for decades which ensures that this mechanism is safe for the human body [3,4]. Chemotherapy for cancer has shown to have various side effects [5]. So, nanoparticles such as SPIONs were introduced to carry drugs to a specific target region [6], which significantly reduces side effects. On the contrary, classical chemotherapy and radiation treatment for cancer cause harm to healthy tissues which is a major drawback. These tiny particles are introduced in the blood stream and navigated to a target region where the cancer cells are located. The drug is locally released to safely destroy the cancerous cells. After that, the SPIONs can be excreted by the body safely. These kinds of experiments have been successfully performed ex vivo and in vivo on animals. Since these particles are superparamagnetic in nature their trajectory can be controlled by magnetic field gradients [7].

Several applications of SPIONs have been achieved in the past decades [8]. Targeted drug delivery, the determination of physical characteristics of SPIONs, how each characteristic plays a role, for example, how can mass affect the magnetic force in the particles, is elucidated. The amount of magnetic force depends on molecular size of the MNPs and the magnetic gradient [9].

The research and usage of SPIONs are widely varied in each field, so learning how to steer them in the desired direction has gained significance. When it comes to navigating SPIONs in blood vessels, the blood vessel is not just one straight tube, it is a complex highly branched network in the body with different diameters in each part of the body. This makes the model more challenging to design the propagation of SPIONs in the fluid flow. In ref. [10], Thalmayer et al. have already investigated how the SPIONs are deflected using a single non moving magnet, what forces are acting upon them, how fast they move in the presence of an external magnetic field. The influences of forces like fluid flow and gravity are also analyzed. Several ongoing research studies are being made on how to create magnetic fields to navigate the SPIONs in human body where these tiny nanoparticles are coated with drugs to be delivered locally into the tumor cells. But the biggest challenge is that the blood vessels are highly branched and to manoeuvre them to the target region involves careful design of the magnetic fields with their gradients [11]. Even the modern lab-on-chips are branched, where the SPIONs have to be delivered to the specific region so the necessity for a diligent control of magnetic fields is to be developed.

## 2. State of the Art and Fundamentals of Particles Steering

SPIONs were first invented in the late 1970s in the field of biomedicine, for the treatment of cancer and mental disease [12]. In the 1990s, SPIONs were further modeled for Magnetic Resonance Imaging (MRI), which opened pathways for nano medicine [13]. In recent times, SPIONs have been extensively researched in the medical fields for drug targeting, where the SPIONs are coated with drugs to destroy cancer cells and they are used for various medical therapies [14].

SPIONs have to be steered to reach the desired direction for drug delivery. In this paper principles of electric motors are used for drug delivery. There are several forms of motors available for applications in the modern world. One important motor which is of significance to this work is the linear motor. The linear motor has a rotor which just moves in one axis, linearly without any rotational motion. This principle follows the version where the propagating magnetic field in the stator of the motor is synchronized with the speed of the rotor velocity, which means the speed with which the magnetic field around the stator moves is equal to the apeed of the mechanical rotation of the rotor. An interesting principle which is used in the entire design of Shanghai’s famous Maglev train. The train has superconducting magnets and the side guidelines have their electric coils, which have the switching currents and thus the resulting propagating magnetic fields. This magnetic fields create the dipole-dipole interactions with the superconducting magnets on the train which propels the train forward [15]. A similar mechanism is used in this current article where a propagating magnetic field is created which is synchronized with the fluid flow in which the SPIONs are released. This means that the speed with which the permanent magnet moves forward is equal to the speed of the SPIONs movement in laminar flow of the fluid. This propagating magnetic field controls the movement of SPIONs and pushes them forward.

### 2.1. Conventional Steering Methods

One of the major navigation methods explored in the past is the one where the patient is injected with SPIONs and using robotic arms the permanent magnets are controlled to steer the SPIONs inside the body [16]. Gradients are now used to navigate inside the human body [17]. This principle of magnetic attraction is studied and is used widely in these methods along with a robotic arm, which moves a single magnet or an electromagnet to guide the SPIONs coated with drugs to a specific region of the body where the tumor is present. The arm control is given importance here which in turn controls the movement of the magnet. The basic formula for magnetic actuation is given by Equation (1) [17].

(1) $$\vec{F_{m}}=\eta .V_{m}.\left(\vec{M}.\nabla \right)\vec{B}$$

where $\vec{F_{m}}$ represents the magnetic actuation force, η is the duty cycle of the pulling force, $V_{M}$ is the total effective volume of magnetic material, B is the magnetic flux density and $\vec{M}$ is the magnetization level of the magnetic material.

Electromagnets are used for magnetic particles steering for decades in the human body but they have certain limitations. The ideas are creative and well tested [18,19] but in this paper, specifically a large number of SPIONs are steered using a series of array electromagnets.

### 2.2. Limitations of Conventional Steering

There are several limitations that must be addressed in the current steering methods. While the robotic arms with a PM is moved for the steering principle is a good technique, a robot near an MRI machine or a magnet near the MRI machine can be potentially dangerous. In one study where a Halbach array is used to steer the SPIONs [20]—which is another novel approach—it has been established that the simulations were performed in 2D designs to speed up simulations. The simulation coverage was defined in the sense that the SPION structuring was limited and that not all the forces affecting their movement were reflected in the model. The agglomeration of SPIONs induced by particle—particle interaction posed another limitation. One significant limitation of therapy is attributed to the fact that SPIONs get lost to the walls of the vessels, say tubes. If the navigation technologies are not precise enough, these MNPs can stick to the walls and the number of SPIONs in the targeted region of a highly branched network becomes insignificant. The trapped SPIONs can also go through the vessel wall into the surrounding tissue. So the drug moves toward the steering magnet and not to the tumor where we want it to be, hence the creation of high-gradient fields for corner navigation is also a challenge.

The limitation of MNPs sticking to the walls can be resolved by using the principle of moving magnetic fields inspired by linear motors and MAGLEV trains.

### 2.3. Inspiration from Linear Motors and MAGLEV Trains

Electric motors, specifically linear motors work on the principle of electromagnetic force or the strong Lorentz force. A linear synchronous motor is a motor where the rotor and the stator speeds are always the same [21]. A MAGLEV train on the other hand to move forward uses the concept of propulsion. In the tracks of a MAGLEV train, there are sequential coils arranged on the tracks. Current is passed alternatively in these coils and the magnetic wave starts moving across the coils creating polarities. The MAGLEV train has superconducting magnets that have north and south poles. These create a constant attraction and repulsion forces which makes the train moves forward [22]. This concept of speed synchronization is explored regarding possibilities of controlling magnetic fields for steering of SPIONs.

## 3. Moving Magnetic Fields

In contrast to a rotational electric motor, a linear motor has a moving magnetic field in the 1D axis direction because of its linear motion [23]. The movement of a magnetic field is similar to propagation of electromagnetic waves. In this paper, we refer to the movement of the magnetic field in multiple ways. One example, when a magnet moves, the magnetic field gets displaced around it in time, which is similar to the movement of the magnetic stator field of the linear motor, when each coil current is turned on sequentially to get the magnetic field around the coils to move in the forward direction creating a propelling force.

### 3.1. Simulation Model in COMSOL Multiphysics

The simulations were performed in COMSOL Multiphysics 6.3 and 6.4. An equal bifurcation channel was designed as shown in Figure 1. In the first simulations a permanent magnet was placed outside the channel and a moving magnetic field is created as the magent moves. The release point has a diameter of 0.1 mm initially and then increased to 0.3 mm. Then an array of magnets is created along the tube and they are modeled as electromagnets which is shown in Figure 3. To study the SPIONs movement again the release region of SPIONs was varied from 0.1 mm and 0.3 mm.

### 3.2. Permanent Magnet Movement for SPIONs Navigation

The study published in 2013 [17], how magnetic steering is executed in a controlled way by a robotic arm to steer the MNPs inside the human body using a permanent magnet. A navigable agent known as a therapeutic magnetic micro-carrier (TMMC) is tested on animal hepatocytes, which is a good breakthrough with a therapeutic micro-carrier to steer MNPs across the human body for drug delivery. In addition to this, magnets with various properties like shape, orientation, magnetic intensity and distancing were selected to control and trap the MNPs [24]. The authors specifically target magnetic energies substantially higher than the Brownian motion.

In the current paper, the concept of linear motors and MAGLEV trains is used to create a moving magnetic field. A horizontal bifurcated microfluidic channel with two branches (upper and lower branch) as shown in Figure 1, is considered to study the SPION movement. A laminar flow is considered in the microfluidic channels for the pure water flow. In a laminar flow of any fluid in a tube, the speed of water is the highest at the center of the tube and the water speed is close to zero at the walls of the tube. When the SPIONs are released at one time, there is a permanent magnet which is moving at the average speed of fluid flow in the center, i.e, the speed of moving magnet and the speed of the fluid flow are synchronized. This allows the SPIONs to be transported to the desired branch with minimal loss of SPIONs either to the walls or to the unintended lower branch. This is similar to the principle of a MAGLEV train which has superconducting magnets and the propulsion force is created because of the speed synchronization of the magnets in the train and the alternating magnetic fields in the coils of the track.

There are two forces which mainly influence the trajectory of the particles. One is the drag force and the other one is the magnetophoretic force. The drag force is given by Equation (2).

(2) $$F_{D}=\frac{1}{\tau_{p}}\;m_{p}\;\left(u-v\right)$$

Drag force formula is taken from the COMSOL Multiphysics document. Here $\tau_{p}$ is the particle velocity response time, $m_{p}$ is the mass of the particle, u is the fluid velocity and v is the particle velocity. The magnetophoretic force is given by Equation (3).

(3) $$F_{m}=2\pi \;r^{3}\;\mu_{0}\;\mu_{r}\;K\;\nabla {\left|H\right|}^{2}$$

Magnetophoretic force formula is also taken from the COMSOL Multiphysics document, where r is the particle radius, $\mu_{0}$ is the vacuum permeability, $\mu_{r}$ is the relative permeability of fluid, K is Clausius-Mossotti factor and H is magnetic field.

For SPIONs to have a desired trajectory, the resultant force of the absolute magnetophoretic force and the absolute drag force should be sufficiently strong to pull the SPIONs in the right trajectory. On the other hand this force should also be so weak that not all of the SPIONs accumulate at the tube wall.

The graph in Figure 2 shows the resultant force due to the drag force and the magnetophoretic force. Especially in the bifurcation of the branch, the net force is directed in the upper branch. The graph and the depiction of forces don’t necessarily have a linear relationship. It is just to show the resultant force is sufficiently high enough to deflect the SPIONs to the upper target branch.

### 3.3. Electromagnetic Array—Working Principle

Electromagnets are explored for magnetic trap based steering [25] by generating a magnetic gradient that allows the particles to steer in a specific location in the human body. But this was explored for permanent magnets named as small scale robots with overcoming just the gravitational force without considering the drag force of the fluid.

There are many other articles [26,27,28,29,30] which talk about various kinds of electromagnet arrangement to control the movement of magnetic nanoparticles all of which include circular or quadrupole arrangement.

In this paper, the magnets are arranged at regular distances, so that when they are turned on sequentially the magnet field moves along with the SPIONs. The speed at which the array electromagnets (EMs) are turned on is synchronized with the center speed of water flow as background media, which means the speed with which the current in the EMs is turned on is equal to the speed of the fluid in the laminar flow. Hence, the same principle of linear synchronous motors is followed here. This speed synchronization creates a sufficient magnetophoretic force which pushes the SPIONs into the intended upper outlet. This again resembles the MAGLEV train, where the train which has superconducting magnets is propelled forward due to the magnetic fields created by the series of electromagnets in the tracks beside them. The speed of the train and the speed with which the currents in the electromagnets are switched on perfectly match in order to create linear motion. Figure 3 depicts this idea of array EMs and the currents switched on sequentially which allows the flow of magnetic fields in the coils.

At any point of time only one magnet is switched on while the others are switched off. Figure 4 shows this as a graph. When the previous magnet switch is turned off, immediately then next magnet switch is turned on.

## 4. Modeling Parameters

Here the particle size is chosen as 500 nano meter to maximize the effect of the magnetic force on the particles which also avoids aggregation [31]. Also the magnetic field intensity is 0.7 Tesla which is the highest when the flow rate is taken as 1 mL/min if it is more then the value also increases.

1. Fluid flow rate: 1 mL/min;
2. Magnet size: 1 mm × 1 mm;
3. Length of the major microfluidic channel: 25 mm;
4. Length of branches: 10 mm;
5. Angle of bifurcation: 60°;
6. Coil material: Copper;
7. Coil current range: [1–5] Amps;
8. Number of turns in coils for EMs: 200;
9. Coil geometry designed in COMSOL: square;
10. Magnetic field strength: 0.7 Tesla;
11. Number of SPIONs injected: 1000;
12. Release diameter of SPIONs: (0.1 and 0.3) mm;
13. Diameter of SPIONs: 0.5 μm;
14. Permeability of SPIONs: 1000.

## 5. Results and Discussion

All simulations are performed in COMSOL Multiphysics 6.4. Initially the simulation was performed using a single bifurcation where there is no influence of magnetic fields. In this case only drag force is present. The expected observation was half SPIONs reach the upper branch and the remaining half reach the lower branch which is seen in the simulations.

### 5.1. Moving Permanent Magnet Results

In the next simulation a permanent magnet is moved at the same speed as the SPIONs speed. The speed synchronization is implemented. There is a single point where the SPIONs are released, and the magnet helps to levitate the particles in the center of the tube, eventually to the upper branch which is the target. There are two forces in play, as introduced in Section 3.1, namely magnetophoretic force(y-directed) and drag force(x-directed). Figure 5 shows the 2D simulation of a permanent magnet as stated before being speed synchronised with the water flow and as a result with the speed of SPIONs.

The SPIONs are minimally scattered which allows the magnet to carry them to the upper branch. This is an ideal scenario. A video of the simulation showing the movement of the magnet and the SPIONs is offered with the Supplementary Materials.

When the diameter, at which the SPIONs are released, increases, in this case SPIONs are more distributed over a large tube cross-section, therefore they are also having a larger velocity range(due to the laminar profile) making them disperse over time and distance, especially there are more SPIONs in regions with a slower background velocity. In this case, all the SPIONs don’t reach the desired upper branch. Some of them are lost to walls and some to the lower branch. In Figure 5 this is seen clearly how SPIONs are scattered which leads to a wastage of them by approximately 15%.

### 5.2. Array Electromagnet Results

An array of electromagnets was designed in COMSOL by spacing them in regular intervals. Their magnetic control of SPIONs was studied. In a similar way, when the SPIONs are released from a single point, all the SPIONs reach the targeted upper branch without getting any of them lost to the walls. The current in the coils of the array electromagnet is switched on sequentially, one magnet at a time, with the same speed as the moving SPIONs, which is a similar concept as the MAGLEV trains. In this case the speed of the moving magnetic field with respect to the SPIONs occurs as a result of currents being sequentially switched on in the coils.

With the next approach, in which the SPIONs are released from a larger diameter of the release point, the SPIONs are scattered and lost to the walls and other branches. Figure 6 shows these ideas with a diameter of the release point: 0.1 mm (upper image) and 0.3 mm (lower image).

Also one more crucial difference that is noticed is the length of tail formation with moving magnet and electromagnetic array with a release of diameter 0.3 mm. With the moving PM we can see the tail formation is very long which leads to the loss of SPIONs. In the array EMs the particles are packetised. The particles in the back are accelerated with the switching on of the EM and the particles in the front experience a force as well which decelerates these particles in the front causing a packetisation. This can be an explanation for the long tail formation in the moving PM than the array EM.

### 5.3. Performance Comparison of SPION Distribution or SPION Steering: Tabulation and Graph

There are some significant differences when it comes to either using a permanent magnet or an array of electromagnets to control the trajectory of SPIONs. One aspect is how many particles are steered into the target branch, which in this case is the upper branch. Another aspect is how many of the particles are lost to the tube walls. The third aspect is about how fast they are reaching the target and how abrupt is their transition to the upper branch. This one is illustrated in graphical form.

#### 5.3.1. Tabulation

From the table and the simulation results, the number of SPIONs lost to the walls are negligible. When the diameter of release is small, we see no particles are lost to the walls. In case of a bigger diameter, there was a small portion of SPIONs lost to walls. In case of array EMs, it was negligible as seen in the Table 1.

#### 5.3.2. Graph—Release of SPIONs from a Radius of 0.1 mm

From the tabulation and the graphs of the results we can see that all the SPIONs released are navigated to the upper branch which is the desired branch. None of the particles are lost to the walls. Speed synchronization helps to prevent this effect. One difference between the array electromagnet and permanent magnet which is prominent is the time taken to reach the desired branch. As the SPIONs are moved faster with the EM array they have less of the chance to drift to the walls compared to the moving PM. This proves that the array EMs when turned off have some residual magnetic flux which causes the SPIONs to be pulled back and when the subsequent EMs are turned on the SPIONs are pulled forward which leads to smaller tail and packetisation. This results in the SPIONs reaching the target faster than a moving PM. Figure 7 and Figure 8 are graphical representations of the SPIONs trajectory with a release diameter of 0.1 mm and 0.3 mm respectively.

#### 5.3.3. Graph—Release of SPIONs from a Radius of 0.3 mm

In the next simulations, represented in Figure 8 where the diameter of the release location was increased, the SPIONs were scattered and the tail formed was long in the case of a moving PM. In case of the array EMs the SPIONs tail formation was smaller, which helped to steer SPIONs to the target branch.

The presented results only reflect a fluid flow rate of 1 mL/min. Some more simulations were performed for an increased fluid flow rate of 2 mL/min and 3 mL/min. The parameters like magnetic field intensity, speed of the moving magnet, sequential switching speed and current in the coils has to be increased according to the fluid flow. For example when the speed of the laminar flow is higher, either the magnetic field intensity has to be increased or the size of the magnet is increased to compensate the effect. However it has to be made sure that the magnet size is not big enough which can attract all the SPIONs close to its poles and as a result can stick to the walls entirely. In case of 0.1 mm release diameter, the SPIONs reaching the target is always 100% at flow rates of 2 mL/min and 3 mL/min. But with a release diameter of 0.3 mm, we get 88% SPIONs reaching the target for both 2 mL/min and 3 mL/min flow rates with an array EM. For the moving magnet we get 79.6% and 71.1% of SPIONs reaching the target for the flow rates of 2 mL/min and 3 mL/min respectively. This says that with array EM trapping SPIONs become much easier as the flow rates keep increasing. But this does not change the core idea about SPIONs steering in concurrence with the principles of linear motor and the MAGLEV trains. The videos of the simulation results for the steering with the permanent magnet and EM array of these are attached as Supplementary Materials.

Regarding the experimental discussion of this theory, testing with halbach array has already been proposed by Thalmayer et al. [6]. It is one of the proofs where halbach array is an efficient way to steer SPIONs, it was developed and tested in the lab in Erlangen.

The results represented in this present article are a representation of a novel steering mechanism. By minimizing the area of release of SPIONs we have proved by simulations that all the SPIONs reach the target region, which is a new concept for steering of SPIONs.

## 6. Limitations of Study

There are some limitations to the study. In this paper not all the forces are included. Gravity is not considered, Brownian motion is considered only up to a certain amount when the diameter of the release location is increased and when the SPIONs reach closer to the walls especially in the case of a moving PM. The two major forces that are included are the drag force and the magnetophoretic force as these are the two strongest ones [32]. In reality the size of the SPIONs is not 100% accurate. As the magnetophoretic force depends on the size of the SPION, the magnitude of the force can vary. The wall of the tube is rather a simplified model. In reality some particles do bounce off the walls and not get stuck entirely to the wall. Molecular dynamics tells us that not all particles stick to the wall [33]. Although, it does not question the presented findings. Beyond that dipole dipole interaction is not considered in the simulation model. The paper mainly aims at the SPIONs being navigated to the wanted and unwanted branch which is independent of the wall—trapped particles.

There are other limitations of this study. The blood vessels are simplified as symmetric tubes. The simulations are performed in 2D geometry to speed up the simulation time. The background fluid flow is water and not blood which has higher viscosity and adds more layers of force. Also there can be interactions with red blood cells which is not included in the current paper. Copper coils are considered and they can have thermal loss which leads to heating up of cells and tissues which can damage them if the heat is very high.

## 7. Conclusions and Outlook

This paper discusses some key insights about how to steer SPIONs in the bifurcated branch. It is seen via simulations that when the magnetic field moves at the same speed as the SPIONs, then maximum number of SPIONs can reach the desired branch. This corresponds to the same principles of the working mechanism of MAGLEV trains and linear motors. A fair comparison of the moved PM and array EM was demonstrated in terms of the magnetic field intensities. Array electromagnet performs better than the moving PM in terms of how fast SPIONs reach the destination and how long the SPIONs are dispersed. The SPIONs are faster when they are controlled with an array EM, however, with a moving PM they are relatively slow. Array EM is the discretized version of the moving PM speed. Array EMs perform better because of more degrees of freedom, which offers better control. The amount of current and the number of windings can also be changed with the user specifications. Since the same magnetic field intensity is considered, a fair comparison is performed.

Compared to conventional steering mechanism which has many limitations and many particles lost to the walls and to the wrong outlets this method has a significant success rate. 100% of the particles reach the upper branch in both EM and PM while when the diameter of the release location is increased, SPIONs are scattered and only 90.6% of SPIONs reach the outlet with array EMs while 85.2% reach the upper branch with a speed synchronized moving PM. This implies that with a 3 times cascaded branch, 75% of SPIONs can be sucessfully steered with an array EM.

In further research there could be more inclusion of forces along with gravity, a full inclusion of Brownian motion since it is considered only partially in this current article and dipole-dipole interactions. The coil currents can be reduced to make it more safe inside the human body. Also, a 3-phase current can be simulated where the SPIONs are pulled away from the wall. With different frequencies of the 3-phase curent the SPIONs are also slowed down to get more control when they are released from a bigger inlet. The beauty of the array electromagnets is this degrees of freedom which allows to control the SPIONs movement in manifold way.

## Figures and Tables

**Figure 1:**
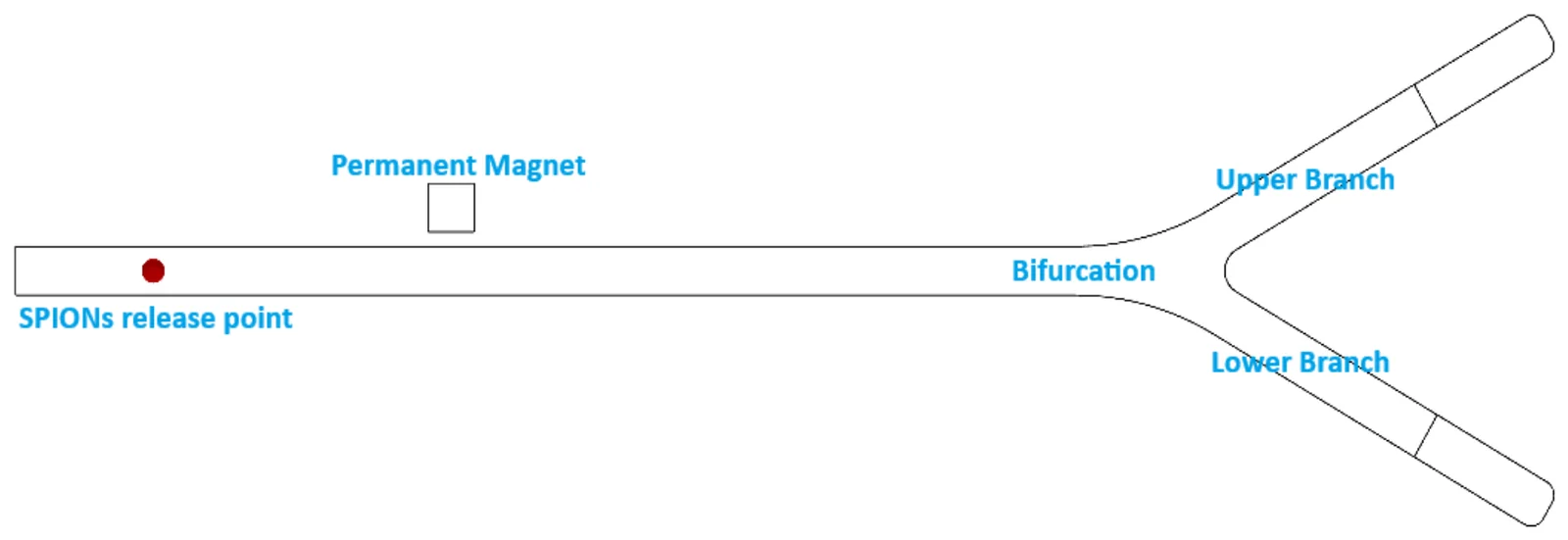
Branches in the models designed.

**Figure 2:**
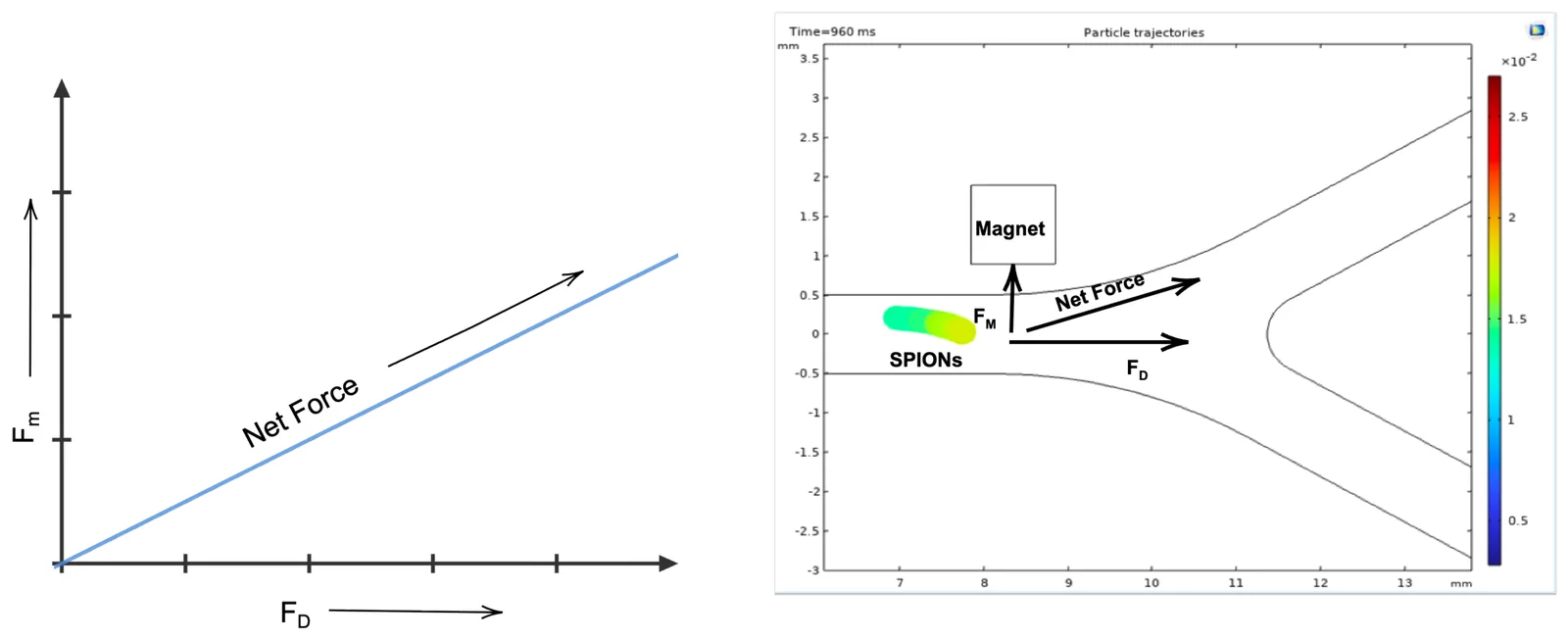
Depiction of forces on SPIONs with the resultant/net force.

**Figure 3:**
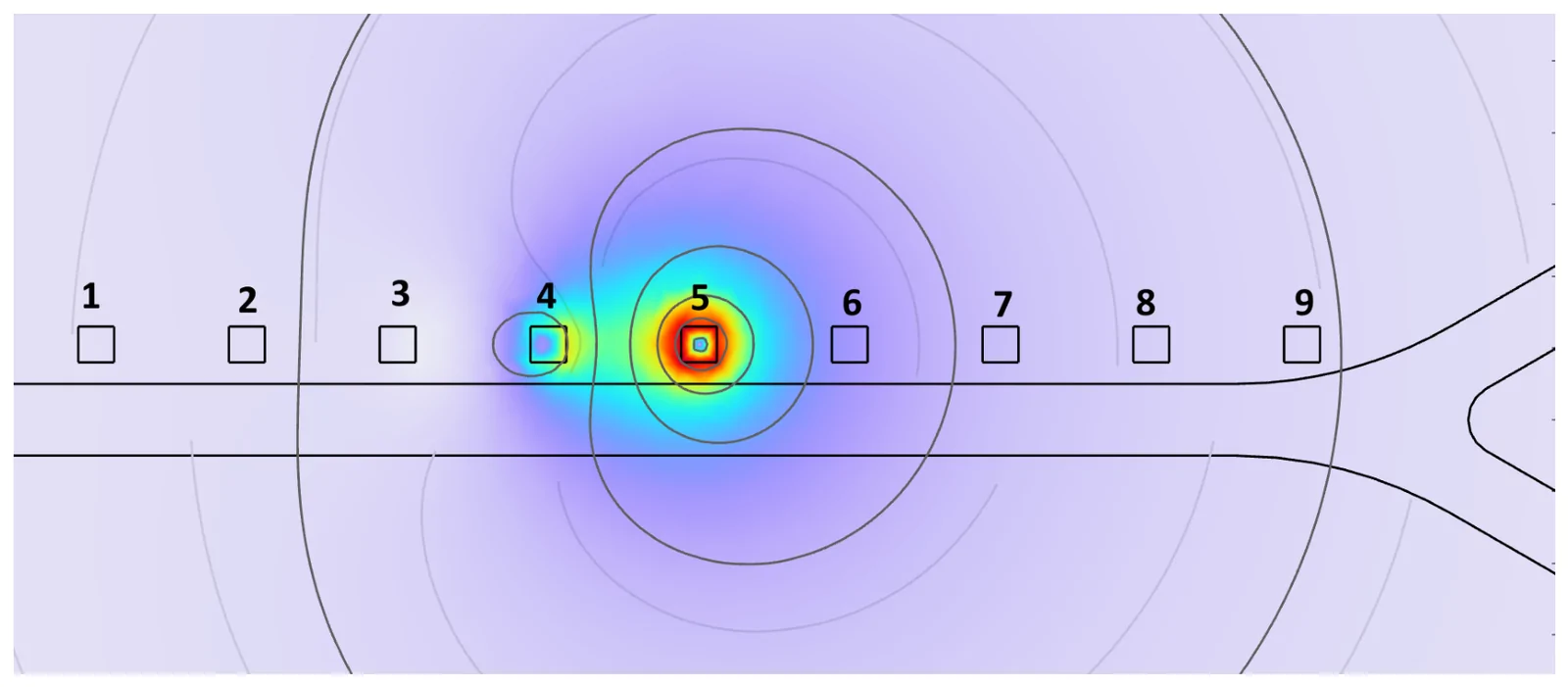
Array electromagnets (represented as small squares) arranged on the side of the tube with the B field when 4th EM is turned off and 5th EM is turned on.

**Figure 4:**
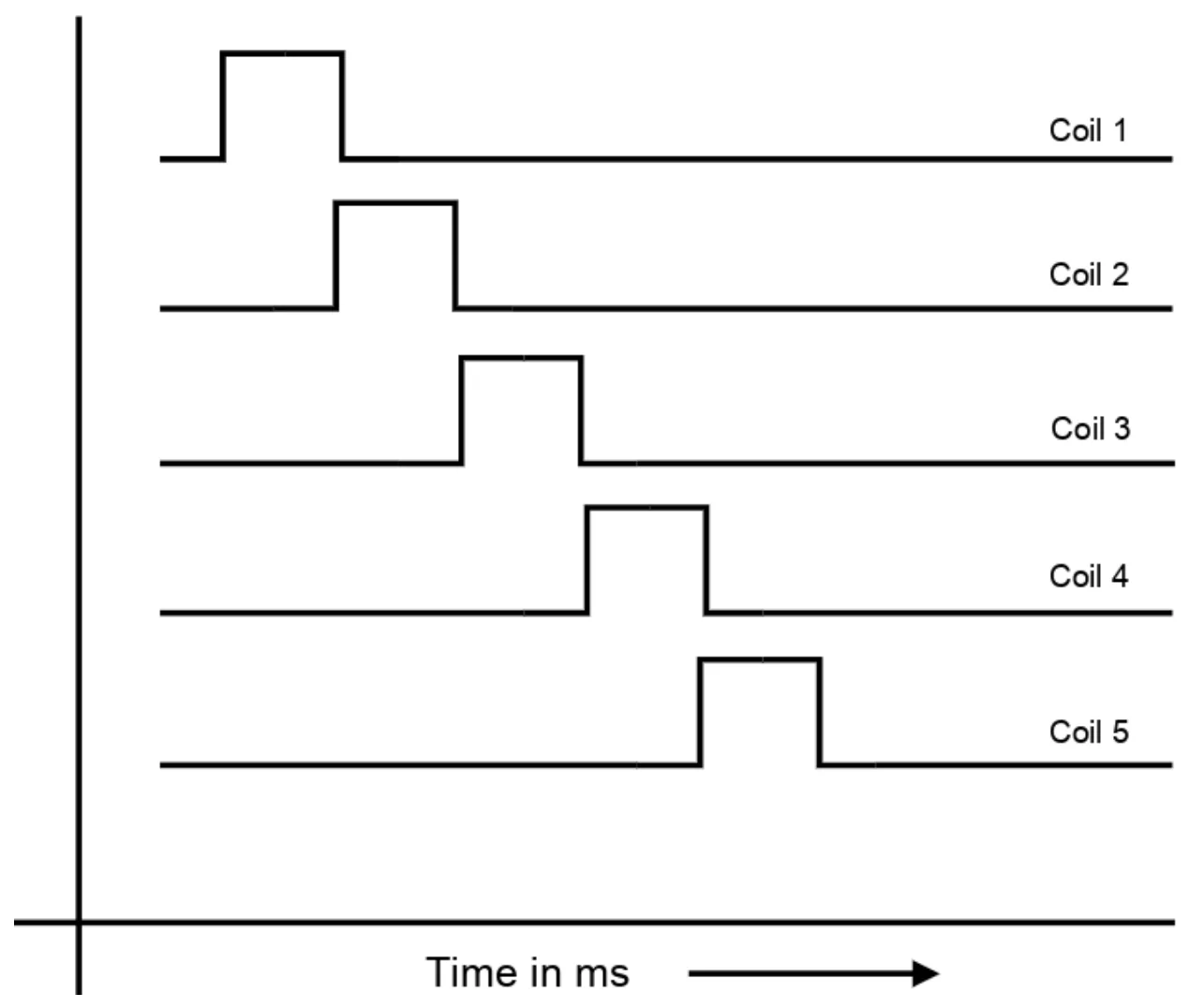
Current switched sequentially.

**Figure 5:**
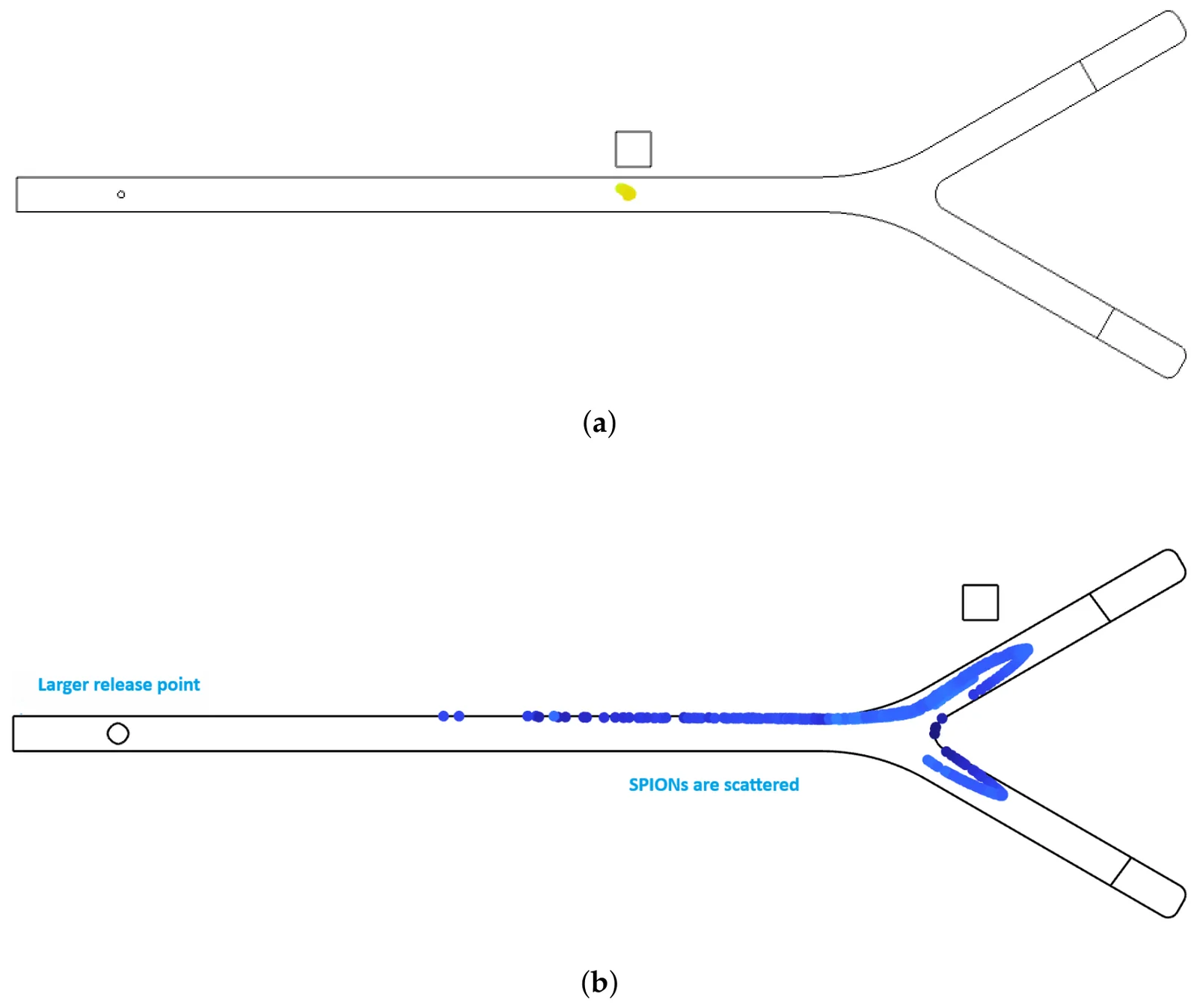
Moving permanent magnet speed synchronized with SPIONs movement. (**a**) SPIONs released from a diameter of 0.1 mm. When the velocity of the background fluid matches the speed with which the magnet moves, then the SPIONs reach the desired outlet, which is the upper branch in the bifurcation. (**b**) SPIONs released from a diameter of 0.3 mm. This shows the SPIONs are scattered and becomes difficult to control getting lost to the walls and bifurcation. There is a huge tail formation of SPIONs in case of a permanent magnet.

**Figure 6:**
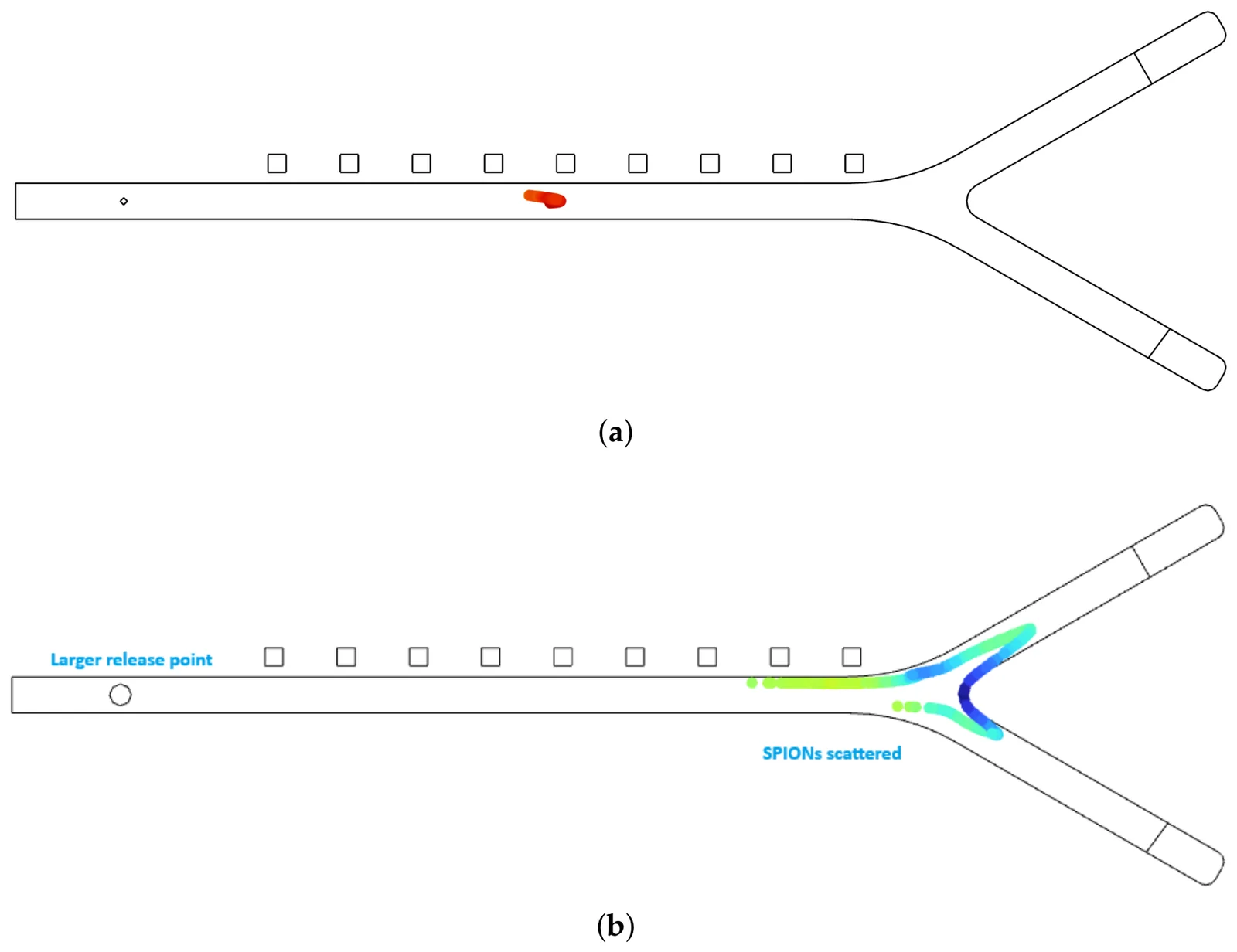
Array electromagnets carrying current sequentially with the speed of SPIONs movement. (**a**) SPIONs released from a diameter of 0.1 mm. The current in the coils are switched according to the speed of the SPIONs travelling in the tube. (**b**) SPIONs released from a diamter of 0.3 mm. On contrary to the moving permanent magnet, switching currents in EMs have smaller tail formation of SPIONs which avoids them getting lost to the walls or the wrong outlet.

**Figure 7:**
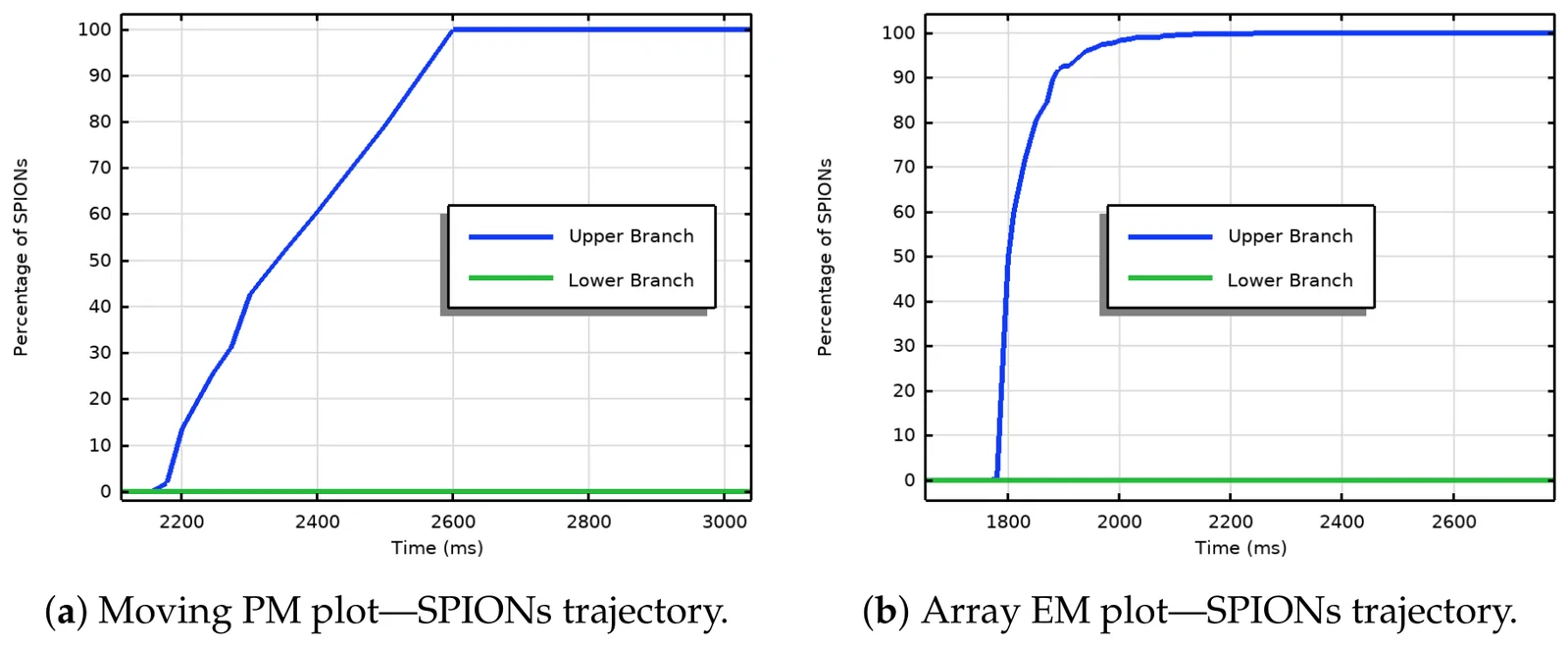
SPIONs released from a diameter of 0.1 mm. The graphs show that all SPIONs reached the right outlet. Also the SPIONs travel faster with EMs than with the PMs. The tail formation is smaller as the transition of the graph is abrupt in Array EM plot.

**Figure 8:**
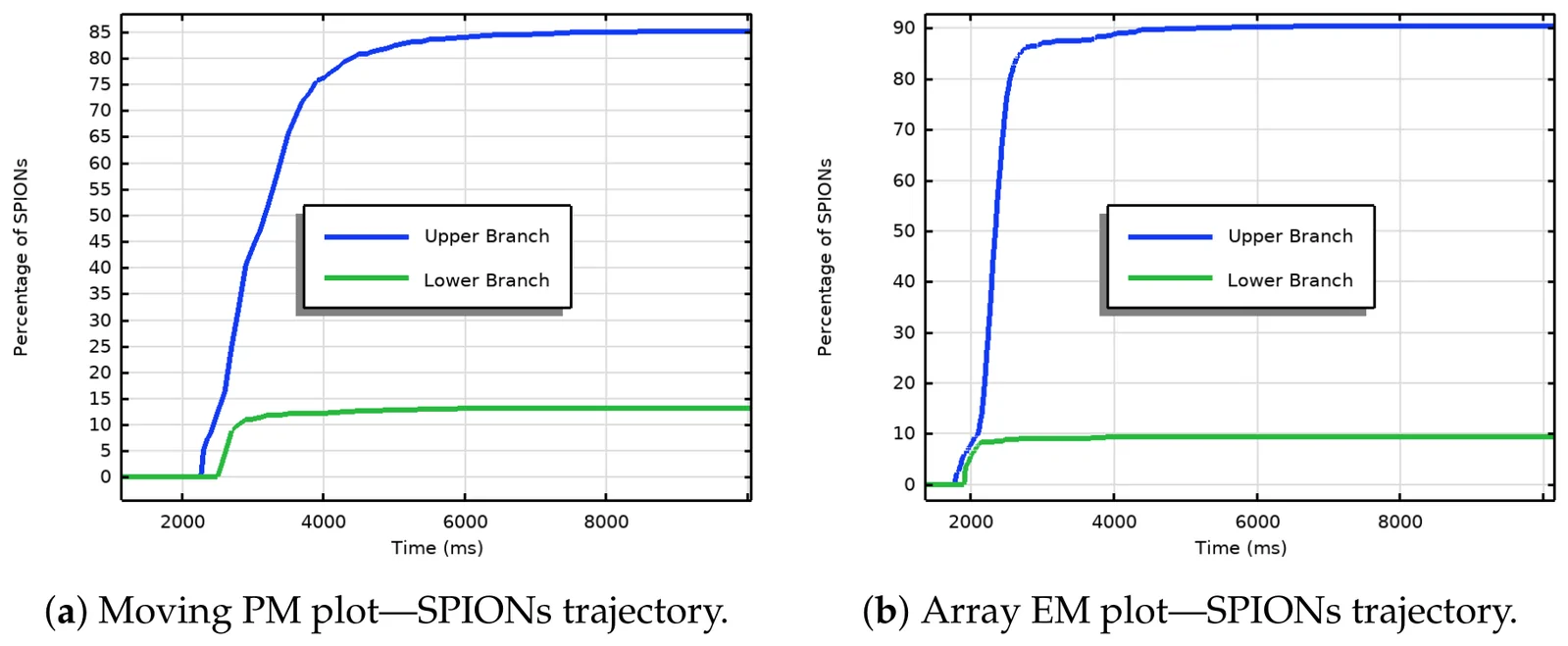
SPIONs released from a diameter of 0.3 mm. From the graph its seen that the amount of SPIONs are more in the target outlet with array EMs rather than the moving PM.

**Table 1:** Simulation results with percentage of SPIONs reaching each outlet.

Type of Magnet	Diameter of Release (mm)	Percentage of SPIONs in Upper Branch (%)	Percentage of SPIONs in Lower Branch (%)	Percentage of SPIONs Lost to Walls (%)
**Moving PM**	0.1	100	0	0
**Array EM**	0.1	100	0	0
**Moving PM**	0.3	85.1	13.3	1.6
**Array EM**	0.3	90.6	9.3	0.1

## Data Availability

There are data attached in the Supplementary Material especially films on SPION propagation.

## Supplementary Materials

The following supporting information can be downloaded at: https://www.mdpi.com/article/10.3390/mi17101125/s1.

## Abbreviations

The following abbreviations are used in this manuscript:

MNP	Magnetic nanoparticle
SPION	Superparamagnetic Iron Oxide nanoparticles
PM	Permanent Magnet
EM	Electromagnet
